# Disclosing quantitative RT‐PCR raw data during manuscript submission: a call for action

**DOI:** 10.1002/1878-0261.13418

**Published:** 2023-03-27

**Authors:** Andreas Untergasser, Jan Hellemans, Michael W. Pfaffl, Jan M. Ruijter, Maurice J. B. van den Hoff, Mihnea P. Dragomir, Douglas Adamoski, Sandra Martha Gomes Dias, Rui Manuel Reis, Manuela Ferracin, Emmanuel Dias‐Neto, Ian Marsh, Mikael Kubista, Muller Fabbri, Ajay Goel, Ondřej Slabý, Erik Knutsen, Baoqing Chen, Massimo Negrini, Koshi Mimori, Martin Pichler, Maria Papatriantafyllou, Simone Anfossi, Thomas D. Schmittgen, Jim Huggett, Stephen Bustin, Jo Vandesompele, George A. Calin, for the HEROIC (tHe initiativE gRoup On qRT dIsClosure) Consortium

**Affiliations:** ^1^ Zentrum für Molekulare Biologie der Universität Heidelberg Germany; ^2^ Genomics Core Facility European Molecular Biology Laboratory (EMBL) Heidelberg Germany; ^3^ CellCarta Zwijnaarde Belgium; ^4^ Division of Animal Physiology and Immunology, School of Life Sciences Weihenstephan Technical University of Munich Freising Germany; ^5^ Department of Medical Biology, Amsterdam UMC, location AMC University of Amsterdam The Netherlands; ^6^ Institute of Pathology, Berlin Institute of Health Charité ‐ Universitätsmedizin Berlin, Corporate Member of Freie Universität Berlin, Humboldt‐Universität zu Berlin Germany; ^7^ Berlin Institute of Health Germany; ^8^ German Cancer Research Center (DKFZ) German Cancer Consortium (DKTK) Heidelberg Germany; ^9^ Brazilian Biosciences National Laboratory (LNBio) Brazilian Center for Research in Energy and Materials (CNPEM) Campinas Brazil; ^10^ Molecular Oncology Research Center Barretos Cancer Hospital Brazil; ^11^ Life and Health Sciences Research Institute (ICVS), School of Health Sciences University of Minho Braga Portugal; ^12^ 3B's‐PT Government Associate Laboratory Braga/Guimarães Portugal; ^13^ Department of Medical and Surgical Sciences (DIMEC) University of Bologna Italy; ^14^ IRCCS Azienda Ospedaliero‐Universitaria di Bologna Italy; ^15^ Laboratory of Medical Genomics Centro Internacional de Pesquisa, AC Camargo Cancer Center São Paulo Brazil; ^16^ Laboratory of Neurosciences (LIM27), Institute of Psychiatry, Faculdade de Medicina Universidade de São Paulo Brazil; ^17^ New South Wales Department of Primary Industries Elizabeth Macarthur Agricultural Institute Narellan Australia; ^18^ TATAA Biocenter AB Göteborg Sweden; ^19^ Institute of Biotechnology CAS, v. v. i. Vestec Czech Republic; ^20^ Center for Cancer and Immunology Research Children's National Hospital Washington DC USA; ^21^ Department of Molecular Diagnostics and Experimental Therapeutics Beckman Research Institute of City of Hope Monrovia CA USA; ^22^ City of Hope Comprehensive Cancer Center Duarte CA USA; ^23^ Department of Biology, Faculty of Medicine, Central European Institute of Technology Masaryk University Brno Czech Republic; ^24^ Department of Comprehensive Cancer Care Masaryk Memorial Cancer Institute Brno Czech Republic; ^25^ Department of Medical Biology, Faculty of Health Sciences UiT‐The Arctic University of Norway Tromsø Norway; ^26^ Department of Radiation Oncology, State Key Laboratory of Oncology in South China, Collaborative Innovation Center for Cancer Medicine Sun Yat‐sen University Cancer Center Guangzhou China; ^27^ Laboratory for Technologies of Advanced Therapies (LTTA), Department of Morphology, Surgery and Experimental Medicine University of Ferrara Italy; ^28^ Department of Surgery Kyushu University Beppu Hospital Japan; ^29^ Division of Oncology, Department of Internal Medicine Medical University of Graz Austria; ^30^ Translational Oncology University Hospital of Augsburg Stenglinstrasse Germany; ^31^ Editorial Manager Molecular Oncology Cambridge UK; ^32^ Department of Translational Molecular Pathology The University of Texas MD Anderson Cancer Center Houston TX USA; ^33^ Department of Pharmaceutics University of Florida College of Pharmacy Gainesville FL USA; ^34^ National Measurement Laboratory LGC Teddington UK; ^35^ School of Biosciences and Medicine, Faculty of Health and Medical Sciences University of Surrey Guildford UK; ^36^ Medical Technology Research Centre Anglia Ruskin University Chelmsford Essex UK; ^37^ Department of Biomolecular Medicine Ghent University Belgium; ^38^ OncoRNALab Cancer Research Institute Ghent (CRIG) Belgium; ^39^ The Center for Non‐codingRNAs University of Texas MD Anderson Cancer Center Houston TX USA

**Keywords:** accuracy, quantification, RNA, RT‐qPCR

## Abstract

Accuracy and transparency of scientific data are becoming more and more relevant with the increasing concern regarding the evaluation of data reproducibility in many research areas. This concern is also true for quantifying coding and noncoding RNAs, with the remarkable increase in publications reporting RNA profiling and sequencing studies. To address the problem, we propose the following recommendations: (a) accurate documentation of experimental procedures in Materials and methods (and not only in the supplementary information, as many journals have a strict mandate for making Materials and methods as visible as possible in the main text); (b) submission of RT‐qPCR raw data for all experiments reported; and (c) adoption of a unified, simple format for submitted RT‐qPCR raw data. The Real‐time PCR Data Essential Spreadsheet Format (RDES) was created for this purpose.

## Graphical abstract

Evaluation of data reproducibility is a growing concern in many research areas. This is especially relevant when examining quantification methods of RNA profiling. The HEROIC consortium members propose to the scientific and medical communities as well as editors to adopt a unified, simple format for submission of RT‐qPCR raw data to journals at the time of manuscript submission.

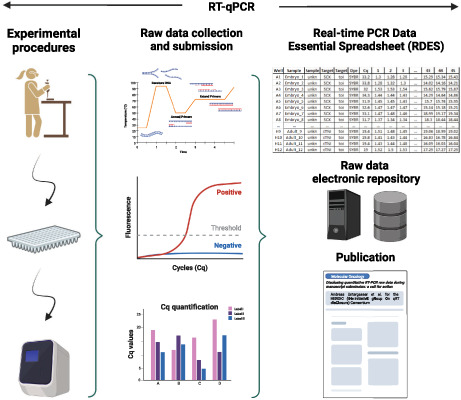

**Abbreviations**
Cq: quantification cycle
GEO: Gene Expression Omnibus
MIAME: minimum information about a microarray experiment
MINSEQE: minimum information about a next‐generation sequencing experiment
MIQE: minimum information for publication of quantitative real‐time PCR experiments
RDES: real‐time PCR data essential spreadsheet
RDML: real‐Time PCR data markup language
RT‐qPCR: reverse transcription‐quantitative polymerase chain reaction

Accurate quantification of coding and noncoding RNAs constitutes an integral part of a multi‐component workflow used to establish differences in gene expression levels among samples in bio‐medical, agricultural, environmental, and industrial research [1]. Reverse transcription‐quantitative polymerase chain reaction (RT‐qPCR) lies at the core of this workflow and has become a ubiquitous method for gene expression analysis. A search of PubMed entries for the words ‘quantitative PCR or real‐time PCR’ in either title or abstract identified about 22 000 papers for 2021 alone, corresponding to ~ 60 publications per day. The past 20 years have witnessed a persistent effort aimed at establishing technical parameters for reliable, reproducible, and biologically meaningful RT‐qPCR experiments [2, 3, 4, 5, 6]. This resulted in the 2009 compilation of the minimum information for publication of quantitative real‐time PCR experiments (MIQE) guidelines [7], as well as in generic requirements for evaluating the performances as described in several International Organization for Standardization documents (such as ISO 20395:2019 or ISO17822:2020). MIQE defines the helpful basic information that should be provided in publications and is necessary for evaluating the technical validity of published RT‐qPCR experiments (especially essential ones such as biomarker development) [8].

However, the quality of most published RT‐qPCR‐based results remains inconsistent, resulting in varying levels of reproducibility and evident concern among researchers, clinicians, journal reviewers, and editors. For example, most published papers provide the reader with no information about RNA purity or integrity [9], RT‐qPCR efficiency [2], detailed amplification conditions, and rationale for chosen normalization strategies. The MIQE guidelines were drafted by scientists to address these exact shortcomings for the benefit of scientists. Moreover, the need to include the PCR efficiency in calculating target quantity, normalized gene expression, or fold‐difference is essential for unbiased reporting of the results of RT‐qPCR experiments [10]. However, reporting quantification cycle (Cq) and PCR efficiency values is insufficient to enable reviewers or readers of a paper to assess bias [11]. Evaluation of the validity of conclusions relying on RT‐qPCR results can be considerably improved if reviewers and readers can examine the amplification curves on which the results were based.

The last two decades have witnessed an increasing concern regarding the evaluation of data reproducibility in many research areas [12]. The conclusions of many assessments, including the most extensively funded and coordinated, the ‘Reproducibility Project: Cancer Biology’ [13], were that more than half of the experiments under scrutiny were not reproduced either in part or totally [14]. Scientists readily acknowledge this to be a major issue. For example, a Nature online survey revealed that about 90% of respondents believed there is a reproducibility crisis in the peer‐reviewed scientific literature, with two‐thirds of respondents experiencing failure to repeat their own results [15, 16]. However, in the case of RT‐qPCR experiments, an essential source of the lack of reproducibility might be the failure to calculate efficiency‐corrected results. Ignoring the assay‐specific PCR efficiency and the inability to standardize the setting of the quantification threshold can lead to significant Cq‐dependent biases in the reported absolute and relative results [17].

One way to tackle this widespread scientific crisis is to start addressing the concerns associated with each component individually. As scientists with wide‐ranging and extensive peer‐reviewed work on the use of RT‐qPCR in the biomedical sciences, we regard the submission of comprehensive RT‐qPCR data as an essential and straightforward step toward addressing this reproducibility crisis. Therefore, we propose to authors, editors, reviewers, publishers, and publication integrity and ethics committees from the biomedical field, as well as the RT‐qPCR equipment producers, the followings:

1. Transparent documentation of the whole experimental process, including factors such as specimen collection, extraction procedure, RNA quality, choice of reverse transcription strategy, oligonucleotide choice (and sequences), and reference gene justification in Materials and methods of a research article, as defined in the MIQE guidelines [7]. Where the laboratory procedures do not change significantly over time, this information can be used for several publications once collected. Successful examples of minimum information that should be included when describing microarray or sequencing studies are the MIAME (Minimum Information About a Microarray Experiment) and the MINSEQE (Minimum Information About a Next‐generation Sequencing Experiment), respectively. In addition, many indexed journals require specific raw data to comply with these standards at the time of submission.
2. Submission of all RT‐qPCR raw data used to generate results reported in a manuscript, ideally at the time of submission to the journal. This will increase the quality of the review, allowing reviewers to assess datasets early on during the peer review process. Furthermore, it will allow editors and editorial staff to analyze data completeness and data integrity even before the initiation of peer review. Alternative options could include requesting RT‐qPCR raw data at the revision step or through preacceptance checklists. These options should be seriously examined by journals and incorporated into editorial workflows as soon as possible.

Technically, we envisage two options for making RT‐qPCR data available. First, raw data could be directly submitted to the journal site. This requires that publishers have in‐house storage capacity and the appropriate security systems to ensure confidentiality while editors and reviewers access raw data to mine the quality and make the data publicly available only upon publication. This will also improve the transparency of data reporting for many journals. Although there is a vast variability in the format of results, depending on the types of analyzed samples (from cell lines with abundant high‐quality RNA to clinical samples from few diseased cells with minute amounts of low‐quality RNA), we support the submission of RT‐qPCR raw data for each experiment included in a specific manuscript. With the expansion of cloud data storage capabilities and given a 384‐well PCR plate generates less than 1 MB of data (usually the amplification data are 150–400 KB and melting curve data 500 KB), we believe the data size issues will be insignificant. Secondly, dedicated data publishing platforms, such as *Scientific Data* (https://www.nature.com/sdata/) or repositories such as figshare (https://figshare.com/) and github (https://github.com), can be used. Data deposited on such platforms could also be cited in the original paper, and the use of data repositories would overcome the need for each journal to create a searchable database of results. For sizeable experiments of at least 20 samples and 20 genes analyzed, RT‐qPCR raw data could be deposited with a public database such as the Gene Expression Omnibus (GEO) database: https://www.ncbi.nlm.nih.gov/geo/info/geo_rtpcr.html.

3 Broad adoption of a simple format of RT‐qPCR raw data for submission to (preferably all) biomedical journals. We present several options to achieve this (see Appendix S1 for the format description and for examples of the format): (a) The use of Real‐time PCR Data Essential Spreadsheet Format (RDES, https://rdml.org/rdes.html; Table 1 as an example). Such files can be created using Microsoft Excel, libreoffice calc software, dedicated tools like RDES‐TableShaper (https://www.gear‐genomics.com/rdml‐tools/tableshaper.html), or a RDES_converter (https://github.com/douglasadamoski/RDES_converter) and contain all the information essentially required for further analysis. As a csv file, it can go to the supplemental files of an article. (b) The XML‐based Real‐Time PCR Data Markup Language (RDML) was developed initially to enable the direct exchange of data and related information between RT‐qPCR instruments and third‐party data analysis software, between colleagues and collaborators and between experimenters and journals or public repositories [18]. We further request that instrument manufacturers implement an option permitting the export of one of the formats in their software. (c) The submission of files according to the requests of the selected database. For example, GEO has specific submission guidelines at https://www.ncbi.nlm.nih.gov/geo/info/geo_rtpcr.html.

**Table 1:** Raw qPCR data in RDES format.^a^

Well	Sample	Sample type	Target	Target type	Dye	Cq	1	2	3	4	…	42	43	44	45
A1	Embryo_1	unkn	SCX	toi	SYBR	33.2	1.30	1.28	1.28	1.27	…	15.10	15.29	15.34	15.43
A2	Embryo_2	unkn	SCX	toi	SYBR	33.8	1.28	1.32	1.30	1.26	…	13.65	14.02	14.16	14.21
A3	Embryo_3	unkn	SCX	toi	SYBR	32.0	1.53	1.53	1.54	1.51	…	15.44	15.62	15.79	15.87
A4	Embryo_4	unkn	SCX	toi	SYBR	34.3	1.44	1.44	1.43	1.42	…	13.81	14.29	14.64	14.86
A5	Embryo_5	unkn	SCX	toi	SYBR	31.9	1.45	1.45	1.43	1.40	…	15.42	15.70	15.74	15.95
A6	Embryo_6	unkn	SCX	toi	SYBR	32.6	1.47	1.47	1.47	1.44	…	15.02	15.14	15.18	15.21
A7	Embryo_7	unkn	SCX	toi	SYBR	33.1	1.47	1.48	1.46	1.45	…	18.54	18.99	19.17	19.34
A8	Embryo_8	unkn	SCX	toi	SYBR	31.7	1.37	1.34	1.34	1.30	…	18.15	18.30	18.44	18.44
…	…	…	…	…	…	…	…	…	…	…	…	…	…	…	…
H9	Adult_9	unkn	cTNI	toi	SYBR	19.4	1.51	1.48	1.49	1.48	…	19.01	19.06	18.99	19.02
H10	Adult_10	unkn	cTNI	toi	SYBR	19.8	1.41	1.43	1.44	1.49	…	16.80	16.83	16.78	16.84
H11	Adult_11	unkn	cTNI	toi	SYBR	19.4	1.43	1.44	1.48	1.47	…	16.13	16.09	16.03	16.04
H12	Adult_12	unkn	cTNI	toi	SYBR	19.0	1.52	1.50	1.53	1.50	…	17.35	17.29	17.27	17.29

^a^ The wells A9–H8 and the cycles 5–41 were left out.

This coordinated effort between scientists, authors, editors, publishers, and equipment producers will pave the way for more data transparency and less erroneous data published. The development of simplified submission tools will be helpful in the near future for raw data deposition from novel technologies with massive expansion at the present time, such as digital PCR or CRISPR genetic screenings. This uncomplicated effort will enhance the RT‐qPCR nucleic acid analysis quality when ever‐increasing demands are being made regarding precision and throughput across the life science sector.

## Conflict of interest

GAC is the scientific founder of Ithax Pharmaceuticals. AU drafted the RDES format and is a member of the RDML consortium. The other authors declare no conflict of interest.

## Supporting information


**Appendix S1.** The Real‐time PCR data essential spreadsheet format (RDES).
