# Supplementary material for: Disclosing quantitative RT‐PCR raw data during manuscript submission: a call for action
Source: Mol Oncol. 2023 Mar 27;17(5):713–7. doi: 10.1002/1878-0261.13418 (PMC10158759; doi:10.1002/1878-0261.13418)
Supplement: Supplementary file 1 — Appendix S1. The Real‐time PCR data essential spreadsheet format (RDES). [file MOL2-17-713-s001.pdf]

The Real-time PCR Data Essential Spreadsheet Format (RDES)

Version 1.0.0

Please check for updated versions at: https://rdml.org/rdes.html

Introduction

Although the Real-time PCR Data Markup Language (RDML) is a comprehensive and advanced format for the documentation, storage and analysis of qPCR data, it may be - due to its XML nature and rich feature set - too overwhelming for some users or applications. The Real-time PCR Data Essential Spreadsheet Format (RDES) was designed as a simple export format for raw qPCR data using classic spreadsheet tables. Furthermore, RDES files can be easily used to create RDML files to which additional information can be added using tools like RDML-Edit (part of RDML-Tools).

Summary

RDES collects the data of only one single run in up to two separate spreadsheet table files, one with amplification data and a second file with melting curve data, if available. In both files the spreadsheet table must use UTF-8 encoding, Linux newline characters (\n) and tabs (\t) as column separators. Dot must be the decimal separator. The file ending should be tsv, but csv and txt are also acceptable. The first seven columns must be Well, Sample, Sample Type, Target, Target Type, Dye and Cq (cycle of quantification) or Tm (melting temperature). The well must be given as letter number combination like A10. The sample column provides the user provided sample names. Sample type must be one of the 3-4 letter codes: unkn (unknown sample), ntc (non-template control), nac (no amplification control), std (standard sample), ntp (no target present), nrt (minusRT), pos (positive control) or opt (optical calibrator sample). Samples with the same sample name in column 2 must have the same sample type in column 3. If sample types are not available, use unkn for column 3. The target column provides the user provided target names. Target Type must be a 3 letter code toi (target of interest) or ref (reference target). If target types are not available, use toi for column 5. In dye the dye name must be given, like SYBR or the measurement frequency like 521 nm. Targets with the same target name in column 4 must have the same target type in column 5 and dye in column 6. The cycle of quantification (Cq) (amplification data) or measured melting temperature value(s) of the amplicon(s) (Tm) (melting data) can be provided in column 7, if available. The column must be present independent of the availability of Cq or Tm values. If the Cq values are not available for individual or for all rows, the cell(s) must be empty. Reactions where the calculation of a Cq value was attempted, but failed, can be indicated with the value -1.0. From column 8 onward the raw data follow. In the first row either the cycle is given as integer (amplification data) or the temperature in degree Celsius is given as floating point number (melting data). Amplification data and melting curve data need to be provided in two independent files. From row 2 and column 8 the measured raw data values follow.

IMPORTANT: The fluorescence values must be raw values as initially measured by the machine and should only be corrected for so-called machine background. It is essential that no correction for baseline fluorescence was performed on this data. Raw fluorescence data should not have negative values.

License

The RDES format and this document are distributed under MIT license.

Details

Experience from the RDML format suggests an improved compatibility if all details are spelled out as explicit as possible.

1. Data Format

1.1. The files must be a plain text file. The file ending should be tsv, but csv and txt are also acceptable.

1.2. The file must use UTF-8 encoding.

1.3. The file must use Linux newline characters (\n).

1.4. The file must use tabs (\t) as column separators. Tabs may not occur in any text within cells of the table. If present, they must be replaced by spaces. Text in cells therefore can not contain tabs and cells may not be enclosed in quotation marks.

1.5. Dot must be the decimal separator. No further indicator for 1000 blocks must be used in numbers.

2. Columns describing the plate layout

2.1. Well: The content of the cell in row 1, column 1 must be Well. In the rows below in column 1 the well must be given as upper case letter + number combination like A10 and resemble the labeling of plates. If qPCR machines do not use a two dimensional setup like for example rotor machines, the positions must be given only as numbers or with a preceding A. Empty wells can be left out, the numbers do not have to be free of gaps. If more than one letter is required, the same number of letters must be used in all wells.

2.2. Sample: The content of the cell in row 1, column 2 must be Sample. In the rows below in column 2 the user provided sample names follow. The names should be human readable.

2.3. Sample Type: The content of the cell in row 1, column 3 must be Sample Type. In the rows below in column 3 the sample type must be given as one of the 3 to 4 letter codes:

unkn - unknown sample

ntc - non-template control

nac - no amplification control

std - standard sample

ntp - no target present

nrt - minusRT

pos - positive control

opt - optical calibrator sample

2.4. Target: The content of the cell in row 1, column 4 must be Target. In the rows below in column 4 the user provided target names follow. The names should be human readable.

2.5. Target Type: The content of the cell in row 1, column 5 must be Target Type. In the rows below in column 5 the target type must be given as one of the 3 letter codes:

toi - target of interest

ref - reference target

2.6. Dye: The content of the cell in row 1, column 6 must be Dye. In the rows below in column 6 a human readable dye description should be provided. If the dye may be not be provided by the user, the measured fluorescence can be given for example as 521 nm.

2.7. Limiting conditions and usage notes

2.7.1. Samples with the same sample name in column 2 must have the same sample type in column 3.

2.7.2. If sample types are not available, use unkn for column 3.

2.7.3. Targets with the same target name in column 4 must have the same target type in column 5 and dye in column 6.

2.7.4. Targets detecting the same gene using different primers or a different dye must use different target names.

2.7.5. If target types are not available, use toi for column 5.

2.7.6. Multiplex data require a separate row for each measured color. In a multiplex reaction, the columns 1-3 are identical and the columns 4-6 must differ to reflect the different targets and dyes.

2.7.7. Technical replicates must use the same sample name and target name. The columns 2-6 will be identical between technical replicates.

3. One column with Cq (amplification data) or Tm (melting data) values

3.1. Amplification data require a column with Cq: The content of the cell in row 1, column 1 must be Cq. Independent of the availability of cycle of quantification (Cq) values the column must be present in all RDES files with amplification data. In the rows below in column 1 the Cq value can be given. If the Cq values are not available for individual or for all rows, the cell(s) must be empty. Reactions where the calculation of a Cq value was attempted, but failed, can be indicated with the value -1.0.

3.2. Melting data require a column with Tm: The content of the cell in row 1, column 1 must be Tm. Independent of the availability of Tm values the column must be present in all RDES files with melting data. In the rows below in column 1 the measured melting temperature value(s) (Tm) of the amplicon(s) can be given. If the Tm values are not available for individual or for all rows, the cell(s) must be empty. If more than one Tm is measured, the individual values must be joined by semicolon (;) without any spaces (example: 82.9;73.6;69.8).

3.3. The decimal separator must be a dot.

3.4. Although this column needs to be present in all RDES files with the correct label in row 1, it is not required to fill it with data.

4. Columns with the fluorescence data

4.1. The first row gives the cycle as integer (amplification data) or the temperature in degree Celsius as floating point number (melting data). For each cycle or temperature a individual column is created.

4.2. From row 2 and column 8 the fluorescence values for the respective cycle or temperature (columns) and well/sample/target (rows) follow.

4.3. IMPORTANT: The fluorescence values must be raw values as initially measured by the machine and should only be corrected for so-called machine background. It is essential that no correction for baseline fluorescence was performed on this data. Raw fluorescence data should not have negative values.

4.4. Amplification data and melting curve data need to be provided in two independent files. The columns 1-6 of these two files are usually identical.

4.5. The decimal separator must be a dot.

4.6. Fractional cycles or more than one measurement per cycle are not supported. The given numbers must reflect the actual cycles. If for example only every second cycle would be measured, the columns would be labeled 1, 3, 5 and so on.
